# Detection of Lower Albuminuria Levels and Early Development of Diabetic Kidney Disease Using an Artificial Intelligence-Based Rule Extraction Approach

**DOI:** 10.3390/diagnostics9040133

**Published:** 2019-09-29

**Authors:** Yoichi Hayashi

**Affiliations:** Department of Computer Science, Meiji University, 1-1-1 Higashimita, Tama-ku, Kawasaki 214-8571, Japan; hayashiy@cs.meiji.ac.jp; Tel.: +81-44-934-7475; Fax: +81-44-931-5161

**Keywords:** diabetic kidney disease, microalbuminuria, normoalbuminuria, cut-off, artificial intelligence, rule extraction

## Abstract

The aim of the present study was to determine the lowest cut-off value for albuminuria levels, which can be used to detect diabetic kidney disease (DKD) using the urinary albumin-to-creatinine ratio (UACR). National Health and Nutrition Examination Survey (NHANES) data for US adults were used, and participants were classified as having diabetes or prediabetes based on a self-report and physiological measures. The study dataset comprised 942 diabetes and 524 prediabetes samples. This study clarified the significance of the lower albuminuria (UACR) levels, which can detect DKD, using an artificial intelligence-based rule extraction approach. The diagnostic rules (15 concrete rules) for both samples were extracted using a recursive-rule eXtraction (Re-RX) algorithm with continuous attributes (continuous Re-RX) to discriminate between prediabetes and diabetes datasets. Continuous Re-RX showed high test accuracy (77.56%) and a large area under the receiver operating characteristics curve (75%), which derived the two cut-off values (6.1 mg/g Cr and 71.00 mg/g Cr) for the lower albuminuria level in the UACR to detect early development of DKD. The early cut-off values for normoalbuminuria (NA) and microalbuminuria (MA) will be determined to help detect CKD and DKD, and to detect diabetes before MA develop and to prevent diabetic complications.

## 1. Introduction

### 1.1. Background

There has been a rapid worldwide increase in the prevalence of type 1 and 2 diabetes mellitus [1], which has caused an increased incidence of diabetic kidney disease (DKD), an intermediate term that can refer to diabetic nephropathy (DN) and chronic kidney disease (CKD) in individuals with diabetes resulting from other causes, such as hypertension [2]. Prediabetes is a transitory stage between normoglycemia and diabetes mellitus that is associated with a high risk of progression to diabetes mellitus; however, the factors underlying this progression remain unclear [3].

The urinary albumin-to-creatinine ratio (UACR) plays a role in predicting the future risk of diabetes mellitus [4]. The UACR was originally introduced into clinical practice to detect incipient DN, as reflected by urinary albumin excretion (UAE), but it has since been shown to be a marker that can predict a variety of important health outcomes, including cardiovascular events, kidney failure, and mortality. However, it is unclear whether a UACR > 30 mg/g Cr is associated with an increased risk of incident diabetes mellitus and CKD-related mortality. The prevalence of normoalbuminuria (NA; UACR < 30 mg/g Cr) in CKD increases with age, with overall means of 9.7% and 4.3% in persons with or without diabetes mellitus, respectively [5].

Although a higher UAE may increase the risk of adverse renal outcomes in persons with diabetes mellitus, it is unclear whether microalbuminuria (MA; UACR 30–300 mg/g Cr) is associated with a higher incidence of macroalbuminuria (UACR > 300 mg/g Cr) in the absence of nondiabetic kidney events that frequently develop over the long-term in type 2 diabetes mellitus. In type 2 diabetes mellitus, a higher UACR, even within the level of NA, has been shown to be associated with a greater incidence of macroalbuminuria when excluding nondiabetic renal events; these findings conflict with those suggesting that MA is a poor indicator of DN progression [6].

### 1.2. Importance of Microalbuminuria

The concept of MA is central to the development of DKD in clinical practice and research. The Kidney Disease: Improving Global Outcomes guideline [7] recommends calculating the UACR to detect proteinuria in patients with or at risk of developing CKD because it has been shown to be a more sensitive indicator of DKD than the protein-to-creatinine ratio [8]. Additionally, urinary albumin assays show a superior analytical performance compared with total protein assays [8]. One of the most common chronic complications in diabetes is DN, so a correct diagnosis is critical. Despite some limitations, urinary albumin level measurement remains the gold standard for diagnosing and categorizing DN because one of the first asymptomatic clinical manifestations of microvascular damage in diabetes is a change in UAE. However, some promising new markers, including those discovered using proteomics-based approaches, have been reported [9].

Several biomarkers have recently been evaluated as potential predictors of renal risk in diabetic patients; however, apart from urinary proteomics, which are expensive, time consuming, and limited in terms of access, none have shown greater performance as a screening tool for DKD than MA, which remains a valid independent predictor for cardiovascular outcomes in diabetes [10].

### 1.3. Study Rationale

The primary rationale behind the present study is that, although no specific UACR threshold has been defined, a continuous relationship exists between UAE and cardiovascular and renal risk and the risk of diabetes. MA is similar to other variables such as fasting plasma glucose (FPG), low-density lipoprotein cholesterol (LDL), and fasting blood pressure (FBG). Additionally, no thresholds for NA or MA have been clearly defined [11], and the categories for defining the UACR seem to have different impacts and predictive values. However, whether such an assumption is correct remains unclear [12]. One of the first studies to address this assumption reported that among women without diabetes, a higher UACR, even within the normal range, was independently associated with an increased risk of developing hypertension, which suggested that the definition of normal UAE needs to be re-evaluated [13].

The most recent American Diabetes Association (ADA) guideline states that, in addition to the UACR being a continuous measurement, differences within normal and abnormal ranges are associated with renal and cardiovascular outcomes [14]. However, the predictive value of a UACR within the normal range for diabetes has not been determined. Therefore, predicting diabetes before the development of MA is expected to facilitate the prevention of diabetic complications [5].

### 1.4. UACR Cut-Off Values for Diseases Associated with Diabetes

Albuminuria was considered to be a continuous variable, so that even in normoalbuminuric type 2 diabetes mellitus patients, UAE rates are correlated with numerous factors that are potentially susceptible to therapeutic intervention; however, correlation does not necessarily imply causation [15]. In their study involving 4449 type 2 diabetes mellitus patients, the UACR was 0.44 mg/mmol (3.89 mg/g Cr).

The prevalence of CKD increased with increasing UACR values and greater numbers of metabolic syndrome characteristics [16]. This, in turn, was associated with a decreased UACR cut-off point and the increased prevalence of CKD. An increased prevalence of CKD among patients with metabolic syndrome was related to UACR values above 10.2 mg/g Cr. However, the study design may have introduced some bias and made it difficult to infer causality between low-grade albuminuria and CKD risk.

The definition of NA for the UACR was arbitrary and did not represent any statistical definition of ‘normal’ [17]. Therefore, the UACR risk level depends on whether this includes the cardiovascular or kidney disease risk. A normal UACR may be described as <10 mg/g Cr in the future, when more accurate DN predictors, markers, and screening tools are developed.

In 2016, Chida et al. [18] reported that patients with a baseline UACR <7.5 mg/g Cr without any pre-existing renal disease were at negligible risk for progression to DN. However, patients with slightly elevated albuminuria (UACR 7.5–30 mg/g Cr) were not considered to be completely free from the risk of developing macroalbuminuria. When incident macroalbuminuria resulting from nondiabetic causes of kidney injury was included in the analysis, a UACR cut-off point of 150 mg/g Cr was the only one that distinguished between neighboring UACR categories. However, these results did not represent the exact incidence of macroalbuminuria in diabetic populations that was associated with some conditions in the cohorts.

To date, many studies have reported UACR cut-off values for diseases that are associated with diabetic diabetes, e.g., MA cut-offs of 19.25 mg/g Cr [19] for hypertension; 10.7 mg/g Cr [20], 15.6 mg/g Cr [21], 10.7 mg/g Cr [22], and 10.0 mg/g Cr [23] for diabetic retinopathy (DR); and 4.8 mg/g Cr [6] for metabolic syndrome. However, only four studies that investigated the cut-off values for type 2 diabetes [15], CKD [16], and DN [17,18] have been conducted.

### 1.5. Study Motivation

Although MA could be a useful marker for progressive CKD and DN, it remains limited. Therefore, while previous studies reported the need for an ‘albuminuric pathway’ [24] to be developed, a prospective study is needed to explicitly explain the mechanism underlying the relationship between albuminuria levels and the risk for both CKD and DN.

Up to one-third of individuals with newly diagnosed diabetes mellitus present some degree of kidney damage, which suggests that the effects of hyperglycemia on the kidney might begin at glycemic levels that are below the diabetic range [25]. However, the long-term influences of prediabetes on the kidney are still unknown. It might be possible for screening for DKD in individuals presenting with prediabetes to lead to early detection and interventions resulting in few new cases of kidney dysfunction [26].

Measurement of FPG and hemoglobin A1c (HbA1c) is an inexpensive and easy way to diagnose diabetes and prediabetes, and it is considered the gold standard. However, this paper focuses on the importance of identifying people before prediabetes develops, using a lower threshold of UACR. This could reduce the use of FPG and HbA1c to detect prediabetes, and could benefit patients by detecting the development of diabetes earlier than currently used methods.

The overall objective for this study was to create an artificial intelligence (AI)-based albuminuric pathway to renal impairment by elucidating the mechanism underlying the relationship between lower NA and MA levels that were used to detect the early development (Stages Ⅰ and Ⅱ) of DKD using rule extraction technology. I hypothesized that the risk of DKD would be found at the intermediate NA creatinine ratio point.

### 1.6. Objectives and Significance

The objectives of the present study were to determine the early cut-off values of NA and MA to help detect CKD and DKD, and to detect prediabetes before NA and MA develop to prevent diabetic complications. The significance of the present study is to develop a new cut-off value to help predict prediabetes, reduce kidney damage and other adverse events that are often associated with prediabetes and kidney disease. This will help patients with prediabetes and kidney disease, and it will also help physicians and healthcare staff who treat these patients to provide the best care possible.

### 1.7. Machine Learning and Rule Extraction

#### 1.7.1. Machine Learning and Rule Extraction

Machine learning is a subfield of AI in which algorithms are trained to perform tasks by learning patterns from data rather than by learning explicit rules [27]. In classic machine learning, experts discern and encode features that appear to be distinctive in the data and use statistical techniques to organize or segregate data based on these features. Statistical analyses of these features in a given image are used to classify or interpret the image. However, for many complex tasks, it is not clear, even to experts, how to define the optimal image features for use in a machine learning algorithm [27].

The rule extraction [28] algorithm in medicine requires many cases and associated final diagnoses as a supervised signal for learning. However, most current diagnostic methods [29,30] for diabetes and prediabetes are ‘black box’ risk-scoring models that do not reveal all the crucial diagnostic information in their risk-scoring models, and thereby remain unclear. For example, even when a diagnosis is correctly assigned to a group, the reason for the classification might not be clear to the physician.

The ‘black box’ nature of machine learning poses considerable problems. A drawback of the black-box models is that they cannot adequately reveal information that may be hidden in the data. For example, even when a method allows the accurate assignment of instances to groups, it is unable to provide the users with the reasoning underlying that assignment. Thus, systems and/or algorithms that can provide insight into these underlying reasons are required [31].

The latest approach in machine learning shows empirical success [32], but substantial concerns remain in terms of its transparency. The inner workings of self-learning machines add an additional layer of complexity and opaqueness to machine behavior. The main task of a machine learning algorithm is to accurately classify all of the data into correct classes. High-performance classifiers have no explainable capability for highly accurate classification. Once a machine learning algorithm is trained, it remains difficult to understand why it provides a particular response to training datasets, and this can be a disadvantage in the medical setting.

This can be a serious disadvantage for these algorithms when used in medical settings because the main task of state-of-the-art machine learning algorithms is to achieve very high accuracy in classifying datasets into separate classes, e.g., diabetes or pre-diabetes. Generally, machine learning algorithms have little capability to explain their classification results.

#### 1.7.2. Objectives of Rule Extraction

The objectives of rule extraction proposed by the present author [31,33] are two-fold. One objective is to reconcile the diagnostic accuracy and interpretability of diagnostic rules to mimic how complex ‘black box’ risk-scoring models make decisions regarding medical diagnoses. However, rule extraction algorithms must extract accurate, concise, and interpretable diagnostic rules for practical use in the medical setting. The other objective is to concisely select and express important and/or influential features of medical datasets in an if–then format. This function often finds the crucial values appearing in an if condition, which are created by the branching of a decision tree such as C4.5 [34]. C4.5 decision trees are classifiers that are characterized by if–then logic that is depicted in a tree structure. They have a high degree of interpretability and are similar to human reasoning.

MA is similar to other variables such as FPG, LDL, and FBG. No thresholds for NA or MA have been clearly defined, and the categories for defining the UACR seem to have different impacts and predictive values. These functions can be used to detect clear cut-offs for lower albuminuria levels in NA and MA. The derived lower NA and MA levels for the UACR and the early development of DKD were observed using an AI-based accuracy-priority rule extraction approach.

## 2. Materials and Methods

### 2.1. Data Source

I used the National Health and Nutrition Survey (NHANES) (1994–2014) dataset [35] (comprising traditional non-invasive factors, blood sampling, laboratory measurements, and demographic and ethnic data) to extract rules for diabetes diagnosis. The NHANES is an ongoing, cross-sectional, probability sample survey of the US population. I limited the study to nonpregnant participants aged 20 years or older.

Participants were considered to have been diagnosed with diabetes if they answered ‘Yes’ to the following question: ‘Have you ever been told by a doctor or health professional that you have diabetes?’ Participants who answered ‘No’ to this question but had a measured FPG level ≥ 126 mg/dL (7.0 mmol/L) and/or hemoglobin A1c (HbA1c) ≥ 6.5% (48 mmol/mol) were considered to have undiagnosed diabetes (Table 1). Participants were considered to have been diagnosed with 1 prediabetes if they answered ‘Yes’ to the following question: ‘Have you ever been told by a doctor or health professional that you have prediabetes?’ Participants who answered ‘No’ to this question but had a measured FPG level between 100 mg/dL (5.6 mmol/L) and 125 mg/dL (6.9 mmol/L) (impaired fasting glucose) and/or an HbA1c level between 5.7% and 6.4% (39–47 mmol/mol) were considered to have undiagnosed prediabetes according to ADA criteria [14].

Participants were excluded if they refused to answer or if they answered ‘Don’t know’ to the above two questions, as were participants with missing or blank data. There are no normoglycemic people in the NHANES diabetes dataset.

Finally, I created a NHANES diabetes dataset comprising 942 (64.3%) diabetes samples and 524 (35.7%) prediabetes samples. The comparison between participants with prediabetes and diabetes is shown in Table 2. Classification models were developed using both types of diabetes samples.

### 2.2. Variable Selection

Because the NHANES is a program that assesses health and nutritional status, the risk factors for diabetic disease in the dataset are common. Although, I considered risk factors in references [36,37,38], I selected 19 variables that are associated with early detection of lifestyle-related diseases (Table 1).

These variables are commonly associated with the risk of diabetes: Two binary (gender and exercise to lose weight), one nominal (race/ethnicity), one ordinal (tobacco use), and 15 continuous (numerical) variables.

### 2.3. Artificial Intelligence with Rule Extraction Technology

#### 2.3.1. The Recursive-Rule eXtraction (Re-RX) Algorithm

The Re-RX algorithm that was recently developed by Setiono et al. [39] was originally designed as a rule extraction tool. The Re-RX algorithm enables a hierarchical, recursive consideration of discrete variables (variables that have only one integer value within a range of values, e.g., sex) before the analysis of continuous data (variables that can have any value within a defined range, e.g., UACR). The Re-RX algorithm eliminates continuous attributes before the C4.5 decision tree is generated using only discrete attributes.

The Re-RX algorithm is a ‘white box’ (more understandable) model that can provide highly accurate and concise classification and be easily explained and interpreted in accordance with the concise extracted rules associated with if–then forms. Therefore, this type of model is often preferred by physicians and clinicians. The Re-RX algorithm provides an accurate rule extraction method that also offers comprehensibility by generating perfect or strict separation between discrete and continuous attributes in the antecedent (if conditions) of each extracted rule.

#### 2.3.2. Re-RX Algorithm with Continuous Attributes (Continuous Re-RX)

The Re-RX algorithm prioritizes the extraction of rules comprising discrete attributes, while continuous Re-RX [31] uses both discrete and continuous attributes to generate the C4.5 decision tree, which results in increased complexity. With mostly continuous input features (variables), we prioritize selecting and expressing important/or influential continuous features in medical datasets that are expressed in an if–then form C4.5 [34].

**Figure 1:**
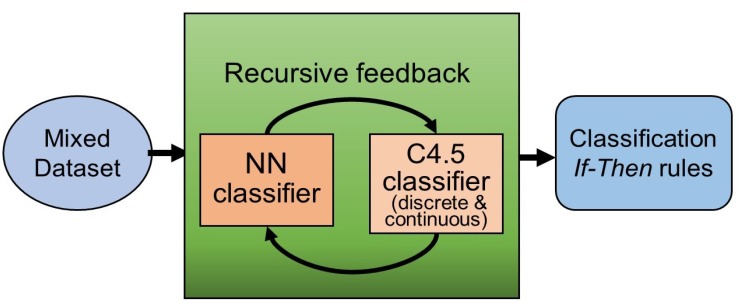
Schematic overview of the recursive-rule eXtraction algorithm with continuous attributes (continuous Re-RX). NN, neural network; C4.5, C4.5 decision tree.

This may seem counterintuitive to the Re-RX algorithm’s design, but the use of both types of attributes also results in increased accuracy. I previously developed the Re-RX family [33], which can deal with various rule extraction situations in the medical setting, meaning that continuous Re-RX belongs to accuracy–priority types [40]. For a better understanding of the mechanism underlying the continuous Re-RX algorithm, a schematic overview is provided in Figure 1.

As shown in Figure 1, the diabetes dataset with all attributes was entered into a neural network (NN) classifier to create a classification model, which was trained by a NN using a backpropagation (BP), and it was then pruned to remove irrelevant and redundant attributes. We then generated a decision tree using C4.5 [34].

We also created if–then rules from the decision tree using post-processing called C4.5 rules [34]. Each rule generated using C4.5 that was not satisfactory for the given criterion was further subdivided (i.e., classification accuracy was enhanced, while the number of attributes per an extracted rule and rules of per a rule set increased) by the Re-RX algorithm [39].

We defined the support for a rule, which is based on the percentage of samples covered by that rule. The support and corresponding error (incorrectly classified) rates of each rule were then checked. If the error exceeded, while the support met, the minimum threshold, then the rule was further subdivided by recursively calling the Re-RX algorithm [39].

**Figure 2:**
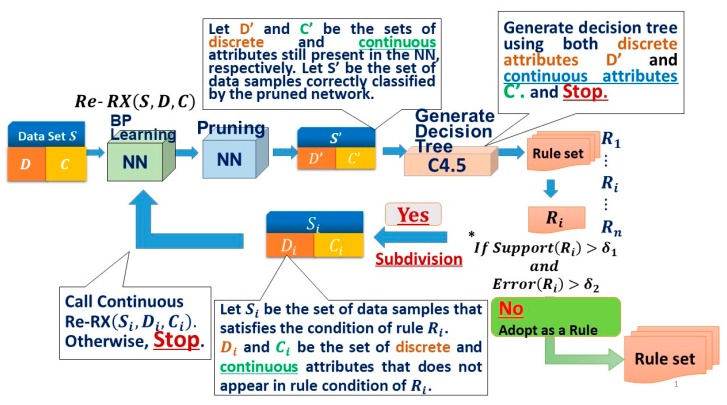
Detailed flow chart of the continuous Re-RX. NN, neural network; C4.5, C4.5 decision tree; D, discrete attributes (variables); C, continuous attributes (variables); BP, backpropagation; δ_1, cover rate;_ δ_2, error rate._

The Re-RX algorithm provides very high classification accuracy and it can also be easily explained and interpreted in terms of concise extracted rules; that is, continuous Re-RX is a ‘white box’ (more understandable) model. I depicted a detailed flow chart of continuous Re-RX in Figure 2.

### 2.4. Performance Measures

To guarantee the validity of the results, k-fold cross validation (CV) [41] was used to evaluate the classification rule accuracy of the test datasets. I trained the dataset using continuous Re-RX and obtained ten runs of five-fold CV accuracies to train the dataset, ten runs of five-fold CV accuracies for the test dataset, the number of extracted rules, and the area under the receiver operating characteristic curve (AUC-ROC) [42] (Table 3). In this paper, the AUC-ROC was used as an appropriate evaluator because it does not include class distribution or misclassification costs [42].

### 2.5. Statistics

To compare clinical parameters between the patients with diabetes and prediabetes, we used a paired *t*-test (*p* < 0.05 was considered significant) for comparisons. The *t*-test is any statistical hypothesis test in which the test statistic follows a Student’s *t*-distribution under the null hypothesis. A *t*-test is most commonly applied when the test statistic follows a normal distribution if the value of a scaling term statistic is known.

## 3. Results

### 3.1. Statistical Comparisons between the Prediabetes and Diabetes Patients

Table 2 shows comparisons of clinical parameters between the patients with prediabetes and diabetes. For diabetes-related factors such as the value of HbA1c, FPG, LDL, race/ethnicity, and UACR, there were significant differences between the prediabetes and diabetes datasets (Table 2).

There are no normoglycemic people in the NHANES diabetes dataset. Table 4 describes the 15 concrete rules that were used to discriminate between prediabetes and diabetes datasets using the AI-based rule extraction approach, which is detailed in Section 3.3. Each condition in a rule is conjunction.

### 3.2. Performance of the Continuous Re-RX Algorithm

I achieved an average accuracy of 77.56% after ten runs of five-fold CV for the NHANES diabetes dataset (Table 3). It took about 61.49 s to train the dataset with the continuous Re-RX using a standard workstation computer (3.1 GHz Intel, 25 MB Cache; 64 GB RAM; 512 GB DDR3 System memory). The testing time was negligible.

This AUC value means that the performance of the predictive model to discriminate between prediabetes and diabetes datasets is high (77.56%). Thus, the predictive model was constructed using the entire set of 15 extracted rules. This shows that the AI-based rule extraction approach can accurately determine the lower albuminuria level (6.1 mg/g Cr). As described in Section 4.3, this paper does not measure the performance of predictive model using the values of FPG and HbA1c.

### 3.3. Rules Extracted Using Continuous Re-RX

#### 3.3.1. Rule 1 and Rule 2

R1 (Rule 1) shows one ethnic parameter and R2 (Rule 2) shows both the ethnic parameters and one critical cut-off for age. Hispanics/Latinos with probable diabetes were young (mean age, 43 years) [43]. This suggests that screening for diabetes needs to start before age 45 years, which is the cut-off recommended by the ADA [44]. In R2, the earlier cut-off for Mexican-Americans is defined as age 40 years, which is close to the mean age of 43 years. About 64% of Hispanics are Mexican-Americans; therefore, R2 is applicable for the prediabetes class. R1 and R2 can be interpreted based on the ADA criteria.

#### 3.3.2. Rule 3, Rule 4, and Rule 5

R3, R4, and R5 can be evaluated based on the ADA criteria.

#### 3.3.3. Rule 6: If HbA1c ≤ 5.6 AND FPG > 122.7 AND UACR ≤ 71.00, Then Class 2 (Prediabetes)

Chida et al. [18] reported that incident macroalbuminuria resulting from nondiabetic causes of kidney injury was included in their analysis, and that 150 mg/g was the only UACR cut-off that was appropriate for discriminating between neighboring UACR categories. Based on the clinical characteristics of such patients by baseline UACR categories, the median was 54.4 mg/g Cr and the interquartile range was 38.1–82.1 mg/g Cr; this median is comparatively close to the present level of 71.0 mg/g Cr.

#### 3.3.4. Rule 8 and Rule 9

R8 and R9 can be evaluated based on the ADA criteria.

#### 3.3.5. Rule 10 and Rule 11

R10 and R11 can be evaluated based on the ADA criteria. The National Diabetes Statistical Report 2016 [44] described racial and ethnic differences in identifying diabetes among adults aged 20 years or older in the US between 2010 and 2012. A higher prevalence of diabetes was seen for both non-Hispanic Blacks (13.2%) and Hispanics (12.8%). ‘Non-Hispanic Blacks = 0: (No)’ comprised all Hispanics except for Mexican-Americans and non-Hispanic Whites.

#### 3.3.6. Rule 11: If HbA1c ∈(6.1, 6.4) AND LDL ≤ 142.0 AND FPG ≤ 108.5 AND

Non-Hispanic Black = 1: (Yes) AND UACR ≤ 6.1, Then Class 2 (Prediabetes)

R11 is the main result of the present paper. Compared with R10, in R11, for non-Hispanic Black = 1: (Yes) AND UACR ≤ 6.1 (mg/g Cr), continuous Re-RX successfully derived the cut-off for NA as 6.1 mg/g Cr, which appears in both R11 and R12.

#### 3.3.7. Rule 13, Rule 14, and Rule 15

R13, R14, and R15 can be evaluated based on the ADA criteria.

## 4. Discussion

### 4.1. Significance of Finding Attributes that are Closely Related to DKD

Lower levels of albuminuria for the UACR were derived as an attribute in accurate and concise if–then rules for discriminating between prediabetes and diabetes datasets using an AI-based rule extraction approach. Lower levels of albuminuria for the UACR appear to be a branching value on the decision tree used in AI-based rule extraction approach. I identified 15 concrete rules by which to discriminate between the datasets. MA is similar to other variables such as FPG, LDL, and FBG, but no thresholds for NA or MA have been clearly defined [11], and the categories for defining the UACR seem to have different impacts and predictive values. The role of urinary albumin, which is closely related to DKD was discussed.

The goal of rule extraction in the clinical setting is to extract accurate, concise, and interpretable rules for medical datasets with mixed and continuous attributes, and to find important and influential discrete and/or continuous attributes for any given dataset without prior knowledge.

The reason that state-of-the-art machine learning approaches cannot find meaningful cut-off values for a given medical dataset is because machine learning only classifies the entire dataset with very high accuracy and is unable to explain the results. However, continuous Re-RX extracts accurate, concise, and interpretable classification rules. The cut-off value of the lowest albuminuria appeared in the attributes of the extracted rules, which can be used for diabetologists and nephrologists. Continuous Re-RX has no substantial limitations on the number of datasets or attributes and runs using a conventional personal computer (PC) within a few minutes.

### 4.2. Significance and Clinical Relevance of This Study

As described in Section 4.3, the extracted rules do not provide new ranges for HbA1c, FPG, and LDL cholesterol to discriminate between prediabetes and diabetes. For the factors on ethnic and age parameters, R6 and R11 demonstrated the upper and lower limit of UACR, which should be noteworthy. However, 71.00 mg/g Cr that was derived in R6 and 150 mg/g Cr was obtained by Chida et al. [18] as the upper limit of UACR are clinically optimistic for early DKD development. In this sense, the most interesting result of this paper is derivation of 6.1 mg/g Cr as the lower albuminuria level for DKD development.

The main result of the present paper is identification of the derived cut-off for NA (6.1 mg/g Cr), which appears in both R11 and R12. A recent study reported that the risk of diabetes increases according to the UAE level, even if it is within the normal range, and that the incidence of diabetes increases in proportion to the UACR [45]. Because we can predict prediabetes before the development of MA, it may be helpful to put effective strategies in place for the prevention of diabetic complications. The rate of diagnosed diabetes for non-Hispanic Blacks is 13.2% [43].

The threshold values of UAE that are associated with increased risk have been reported to be substantially lower than the currently recommended level for MA [46]. Despite a lack of outcome studies, the available data suggest that these limits should be reduced by approximately three-fold, from 30 to 10 mg/g Cr for the UACR.

Park et al. [6] analyzed the relationship between normal range albuminuria and metabolic syndrome in calculating the UACR (4.8 mg/g Cr). The values of 3.89 and 4.8 mg/g Cr in those two studies were relatively close to the value of 6.1 mg/g Cr that was found in the present study. Heo et al. [16] reported that UACR values above 10.2 mg/g Cr were associated with an increased prevalence of CKD among individuals with metabolic syndrome.

Chida et al. [18] found that individuals with a baseline UACR < 7.5 mg/g Cr without any pre-existing renal disease were at negligible risk for progression to DN. The value of 7.5 mg/g Cr obtained in their study is close to the value of 6.1 mg/g Cr found in the present study. Therefore, the range of UACR ≤ 6.1 mg/g Cr can be quite small values, which suggests that R11 is applicable for the diagnosis of prediabetes.

The confounding factor of MA has a ten-times wider range for definitions, e.g., UACR 30–300 mg/g Cr, and thus can allow the detection of various diseases associated with diabetes, because it was designed for multi-purpose use. To find the cut-offs for various diseases associated with diabetes, previous studies [13,15,16,18,19,20,21,22] analyzed AUC-ROCs and used multivariate logistic regression analysis. However, continuous Re-RX can detect the UACR cut-off values for an indication of early DKD and various diseases that are associated with diabetes, without prior knowledge such as distributions/or assumptions of medical datasets.

Important associations have been identified between progressive DKD and the quantity of albumin excreted in the urine [24]. Because the lower levels for the UACR in the diabetes dataset are based only on changes in albuminuria within the NA and MA ranges, this should be interpreted conservatively and viewed only as hypothesis-generating. Additionally, albuminuria is well known to have substantial biological (intraindividual) variation. The accuracy–priority rule extraction approach can be algorithmically extended to a larger cohort of patients with DKD and diseases that are associated with diabetes to derive lower NA and MA levels for the UACR without loss of generality.

I also emphasized that the present AI-based rule extraction approach is different from other clinical studies and methods that are being used for similar research in diabetes, prediabetes, CKD and DKD, such as the use of clinical scores and multiple biomarkers.

However, this study had some limitations. The diabetes dataset consisted of self-reported data, and a relatively small number of samples was used for the modelling approach. We should also investigate patients with type 1 diabetes. These issues are closely related to the cohort design and there is a limited number of patients with type 1 diabetes in the NHANES database. These are important topics for future research.

### 4.3. Significance of This Study for Point-of-Care Clinical Relevance

The dipstick testing for proteinuria was both sensitive and specific for macroalbuminuria, but this was not the case for MA. Among people with diabetes mellitus, dipstick testing cannot replace the dry chemistry-based dipstick methods of screening by directly assessing albuminuria [47]. A specific nationwide health check-up and guidance system, called Tokutei Kenshin, does not include a UACR test.

In Japan, the cost of measuring the UACR and total urine protein-to-creatinine ratio (TPCR) is more than ten-fold higher than that of the TPCR. There was a strong positive correlation between the UACR and TPCR. The optimal cut-off value for the TPCR based on the ROC curve analysis for predicting MA was 84 mg/g Cr (8.4 mg/mmol) [48]. This value is much less sensitive than the lowest albuminuria of MA using a UACR test and the lowest values of NA and MA are difficult to detect.

A study [49] in Australia found that the point-of-care (POC) testing for UACR was less costly (−11.6%) compared with laboratory UACR testing. To confirm the results in Ref. [49], I tabulated Table 5 for comparison of the cost of a laboratory UACR test and a semi-quantitative UACR test in Japan, UK and the US.

Although the cost of POC testing for a semi-quantitative UACR testing is higher than a TPCR test, the POC testing for a semiquantitative UACR have several advantages over laboratory UACR testing, which are as follows: There are no time-consuming sample logistics; the results are obtained more quickly; and the findings can be discussed with the patient during the clinic visit with no further action required if there is a negative result. Moreover, POC tests for semiquantitative UACR can change the care process by enabling faster decision-making and reducing the need for further consultations [52].

An important point in this paper is that the two lower albuminuria levels from UACR that are used to detect DKD, i.e., 6.1 mg/g Cr derived in this paper and 7.5 mg/g Cr [18], are considerably lower than the current lowest albumin level in MA. Thus, even if the current POC test for UACR devices cannot detect considerably lower levels of albuminuria compared with the lowest MA value, i.e., 30 mg/g Cr, the values detected by the POC testing for semi-quantitative UACR will provide earlier identification of potential DKD patients in the diabetic and prediabetic populations whose UACR will increase to a higher level of MA in near future. The lower albuminuria level of 6.1 mg/g Cr provides an AI-based rationale to accept a considerably lower albuminuria level for MA without statistical analysis.

The need to detect the early development of DKD is becoming an important issue for public health. Thus, we should utilize semi-quantitative UACR for POC tests. I believe that there is a need to establish a new transparent AI-based approach to detect early DKD development that is both accurate and inexpensive.

The results presented here suggest the usefulness of adopting lower cut-offs for UACR to detect NA and MA for the prediabetic patient population. Further justification is needed to increase awareness of DKD and ensure that patients have an annual POC test for semi-quantitative UACR or laboratory UACR, which could reduce harm for DKD patients in diabetic and prediabetic populations

## 5. Conclusions

To the best of my knowledge, this is the first study to use transparent AI-based rule extraction technology to determine substantially lower UACR levels for NA and MA for the early detection of CKD and DKD. Because NA and MA are of particular clinical significance in prediabetes, the ability to detect kidney damage in prediabetic patients before NA and MA develop may help to prevent further diabetic complications. The lower UACR levels determined in this study could allow the earlier treatment of kidney damage while it is still at a reversible stage.

## Figures and Tables

**Table 1:** Summary of the National Health and Nutrition Survey (NHANES) diabetes dataset and the definitions of diabetes and prediabetes.

Attribute	Possible Values or Ranges	
Age (years)	20–80+	Continuous
Sex	Male, Female	Binary
Race/ethnicity	Mexican-American Other Hispanic Non-Hispanic White Non-Hispanic Black Other race	Nominal
Systolic blood pressure (mmHg)	62–228	Continuous
Diastolic blood pressure (mmHg)	0–118	Continuous
Waist circumference (cm)	40.2–177.9	Continuous
Body mass index (kg/m^2^)	12.1–82.9	Continuous
Total cholesterol (mg/dL)	69–813	Continuous
Urine albumin-to-creatinine ratio (mg/g Cr)	0.21–9600	Continuous
Glycohemoglobin (%)	3.5–17.5	Continuous
Tobacco use	Every day; some days; not at all	Nominal
Alcohol (average no. alcoholic drinks/day)	1–25	Continuous
Exercise to lose weight	No, Yes	Binary
Triglycerides (mg/dL)	13–4233	Continuous
LDL-cholesterol (mg/dL)	14–375	Continuous
Direct HDL-cholesterol (mg/dL)	8–138	Continuous
Fasting plasma glucose (mg/dL)	51–421	Continuous
Insulin (μU/mL)	0.14–682.48	Continuous
Total bilirubin (mg/dL)	0.1–7.1	Continuous

The following terms were defined: Diabetes, fasting plasma glucose (FPG) ≥ 126 mg/dL (7.0 mmol/L); fasting, no caloric intake for at least 8 h or hemoglobin A1c (HbA1c) ≥ 6.5% (48 mmol/mol); and prediabetes, FPG 100–125 mg/dL (5.6–6.9 mmol/L; impaired fasting glucose) or HbA1c 5.7–6.4% (39–47 mmol/mol). All tests were performed in a laboratory using the method of the National Glycohemoglobin Standardization Program certified and standardized to the Diabetes Control and Complications Trial assay. NHANES, National Health and Nutrition Examination Survey; LDL, low-density-lipoprotein; HDL, high-density-lipoprotein.

**Table 2:** Comparison of participants with prediabetes and diabetes from the NHANES diabetes dataset.

Attribute	Prediabetes (SD)	Diabetes (SD)	p-Value
Age (years)	55.04 (15.79)	60.01 (13.54)	<0.0001
Sex	Male (1): 45.80% Female (0): 54.19%	Male (1): 50.74% Female (0): 49.25%	0.0069
Race/ethnicity	Mexican-American: 13.74% Other Hispanic: 6.87% Hispanic: 9.235% Non-Hispanic White: 51.33% Non-Hispanic Black: 18.51% Other race, including multiracial: 9.54%	Mexican-American: 21.54% Other Hispanic: 9.235% Non-Hispanic White: 38.85% Non-Hispanic Black: 23.03% Other race, including multiracial: 7.32%	<0.0001
Systolic blood pressure (mmHg)	125.47 (18.10)	130.57 (20.15)	<0.0001
Diastolic blood pressure (mmHg)	69.18 (13.14)	68.15 (14.99)	0.0086
Waist circumference (cm)	104.66 (15.75)	107.73 (15.73)	<0.0001
Body mass index (kg/m^2^)	30.95 (7.05)	31.74 (6.93)	0.0018
Total cholesterol (mg/dL)	198.19 (40.73)	183.31 (40.37)	<0.0001
Urine albumin-to-creatinine ratio (mg/g Cr)	8.85	14.85	0.337
Glycohemoglobin (%) HbA1c	5.92 (1.05)	7.38 (1.81)	<0.0001
Tobacco use	Every day: 14.88% Some days: 1.90% Not at all: 83.20%	Every day: 14.33% Some days: 2.22% Not at all: 83.43%	0.88
Alcohol (average # alcoholic drinks/day) median	1	1	0.002
Exercise to lose weight	Yes: 30.34% No: 69.65%	Yes: 26.64% No: 73.35%	0.13
Triglycerides (mg/dL)	134.76 (68.22)	145.92(71.38)	0.0001
LDL-cholesterol (mg/dL)	118.79 (36.29)	104.18 (34.82)	<0.0001
Direct HDL-cholesterol (mg/dL)	52.47 (14.88)	49.93 (13.63)	<0.0001
Fasting plasma glucose (mg/dL) median	108.0	139.8	<0.0001
Insulin (mU/mL) median	12.1	12.74	0.21
Total bilirubin (mg/dL)	0.730 (0.28)	0.72 (0.28)	0.20

**Table 3:** Accuracies after CV for the NHANES diabetes dataset.

NHANES Diabetes Dataset	TR ACC (%)	TS ACC (%)	# Rules	AUC-ROC (%)
Continuous Re-RX [10 × 5 CV]	79.65 ± 0.62	77.56 ± 2.19	15.82	75

CV, cross-validation; Re-RX, recursive-rule eXtraction; continuous Re-RX, Re-RX algorithm with continuous attributes; TR, training dataset; TS, testing dataset; ±, standard deviation; ACC, accuracy; AUC-ROC, area under the receiver operating characteristic curve; 10 × 5 CV, 10 runs of five-fold cross-validation.

**Table 4:** Fifteen concrete rules that were used to discriminate between prediabetes and diabetes datasets using the artificial intelligence (AI)-based rule extraction approach.

IF Part	Condition 1	Condition 2	Condition 3	Condition 4	Condition 5	THEN
R1	HbA1c ≤ 5.8	FPG ≤ 122.7	LDL ≤ 101.0	Mexican-American = 0 (No)		Class 2 (Prediabetes)
R2	HbA1c ≤ 5.8	FPG ≤ 122.7	LDL ≤ 101.0	Mexican American = 1: (Yes)	AGE ≤ 40	Class 2 (Prediabetes)
R3	HbA1c ≤ 5.8	FPG ≤ 122.7	LDL ≤ 101.0	Mexican-American =1: (Yes)	AGE > 40	Class 1 (Diabetes)
R4	HbA1 ∈ (5.8, 6.1)	FPG ≤ 122.7	LDL ≤ 101.0			Class 1 (Diabetes)
R5	HbA1c ≤ 6.1	FPG ≤ 122.7	LDL > 101.0			Class 1 (Diabetes)
R6	HbA1c ≤ 5.6	FPG > 122.7	UACR ≤ 71.00			Class 2 (Prediabetes)
R7	HbA1c ≤ 5.6	FPG > 122.7	UACR > 71.00			Class 1 (Diabetes)
R8	HbA1c ∈ (5.6, 6.1)	FPG > 122.7	LDL ≤ 151.0			Class 1 (Diabetes)
R9	HbA1c ∈ (5.6, 6.1)	FPG > 122.7	LDL > 151.0			Class 2 (Prediabetes)
R10	HbA1c ∈ (6.1, 6.4)	LDL ≤ 142.0	FPG ≤ 108.5	Non-Hispanic Black = 0: (No)		Class 1 (Diabetes)
R11	HbA1c ∈ (6.1, 6.4)	LDL ≤ 142.0	FPG ≤ 108.5	Non-Hispanic Black = 1: (Yes)	UACR ≤ 6.1	Class 2 (Prediabetes)
R12	HbA1c ∈ (6.1, 6.4)	LDL ≤ 142.0	FPG ≤ 108.5	Non-Hispanic Black = 1: (Yes)	UACR > 6.1	Class 1 (Diabetes)
R13	HbA1c ∈ (6.1, 6.4)	LDL ≤ 142.0	FPG > 108.5			Class 1 (Diabetes)
R14	HbA1c ∈ (6.1, 6.4)	LDL > 142.0				Class 2 (Prediabetes)
R15	HbA1c > 6.4					Class 1 (Diabetes)

FPG, fasting plasma glucose; LDL, low-density lipoprotein; UACR, urine albumin-to-creatinine ratio.

**Table 5:** Comparison of the cost of a laboratory UACR test and a semi-quantitative UACR test in several countries.

	Japan	UK	The US
Cost of laboratory test for UACR	JPY 1080 [50]	₤7.4 [51]	$16 [48]
Cost of a POC testing for a semi-quantitative UACR	JPY 230 [50]	₤2.31 [51]	---

UACR: Urine albumin-to-creatinine ratio; POC: point of care.
